# Supplementary material for: Another Move towards Bicalutamide Dissolution and Permeability Improvement with Acetylated β-Cyclodextrin Solid Dispersion
Source: Pharmaceutics. 2022 Jul 15;14(7):1472. doi: 10.3390/pharmaceutics14071472 (PMC9316079; doi:10.3390/pharmaceutics14071472)
Supplement: Supplementary file 1 [file pharmaceutics-14-01472-s001.zip › pharmaceutics-1804159-supplementary.pdf]

Supporting Information

Another move towards bicalutamide dissolution and permeability improvement with acetylated β-cyclodextrin solid dispersion

Tatyana V. Volkova, Olga R. Simonova, German L. Perlovich*

G.A. Krestov Institute of Solution Chemistry RAS, 153045 Ivanovo, Russia
**Correspondence: glp@isc-ras.ru*

Supporting Information - content:

Figure S1. The absorption spectra of BCL in pure buffer pH 6.8 (blue), Me-β-CD (orange), Ac-β-CD (red), n-hexane (green); ethanol (wine). P2

Figure S2. PXRD patterns of BCL raw (a), BCL after solubility experiments with Me-β- (b) and Ac-β- (c) cyclodextrins in pH 6.8 solutions. P3

Figure S3. The temperature dependences of the stability constants for the complexation of BCL with Me-β-CD and Ac-β-CD. P4

Figure S4. Job's plot for BCL/Ac-β-CD inclusion complex at 298.15 K and pH 6.8. P5

Table S1. BCL experimental solubility (S_2) in buffer pH 6.8 with different Me-β-CD and Ac-β-CD concentrations (S_{CD}) in the temperature range of 298.15 - 313.15 K. P6

Table S2. Donor solution BCL concentrations (C), steady state flux (J), and permeability coefficients (P_{app}) of BCL raw sample (BCL_raw), BCL physical mixture (BCL/Ac-β-CD_pm) and ground complex (BCL/Ac-β-CD_gr) with Ac-β-CD, 37 ^{o}C. P6

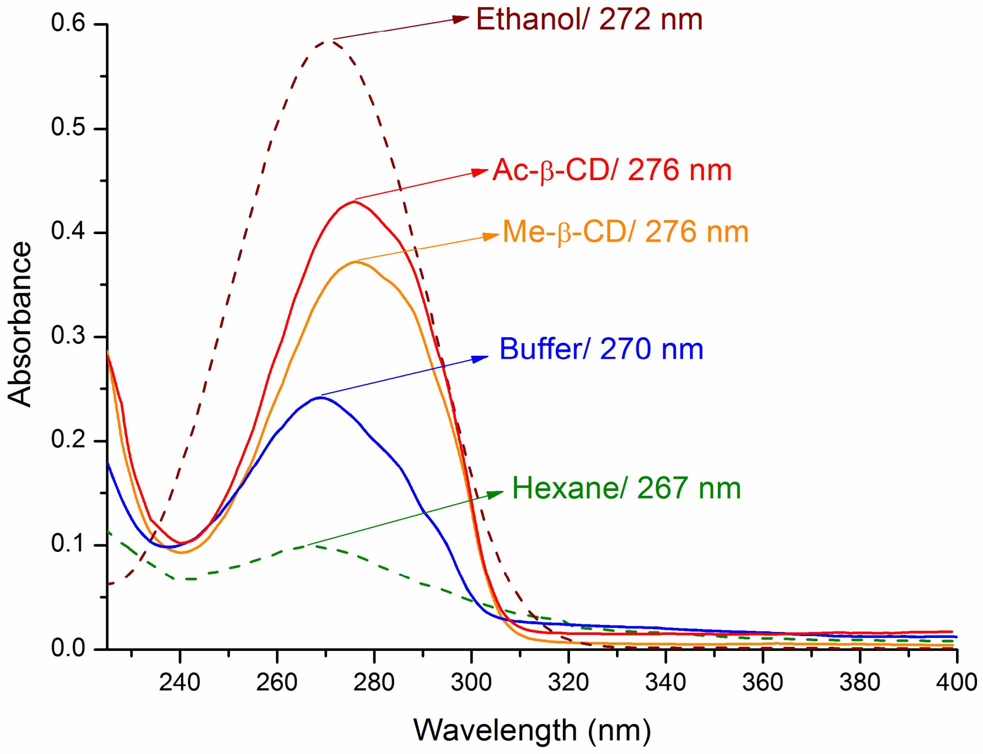

Figure S1. The absorption spectra of BCL in pure buffer pH 6.8 (blue), Me-β-CD (orange), Ac-β-CD (red), n-hexane (green); ethanol (wine).

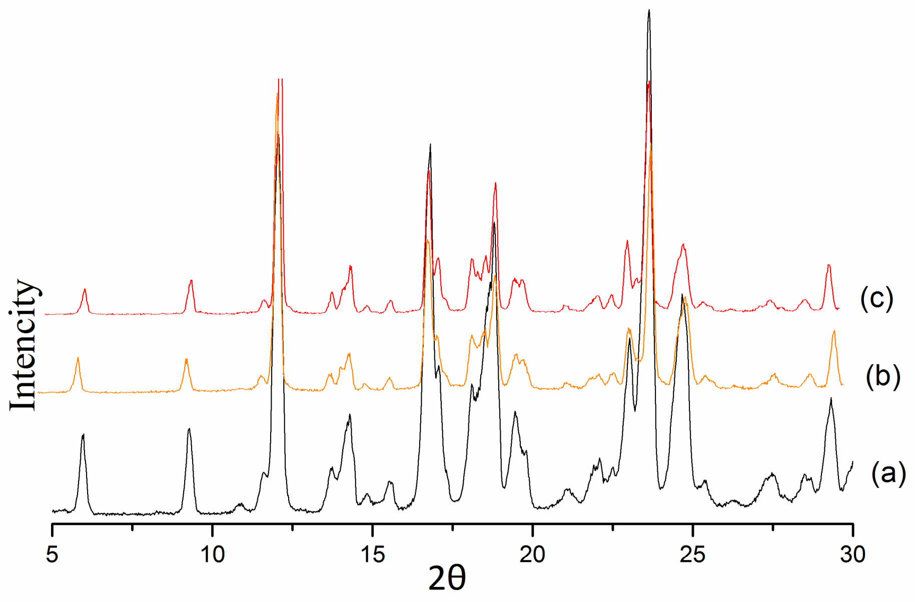

Figure S2. The PXRD patterns of parent BCL (a) and solid residuals after the solubility experiments with Me-β- (b) and Ac-β- (c) cyclodextrins.

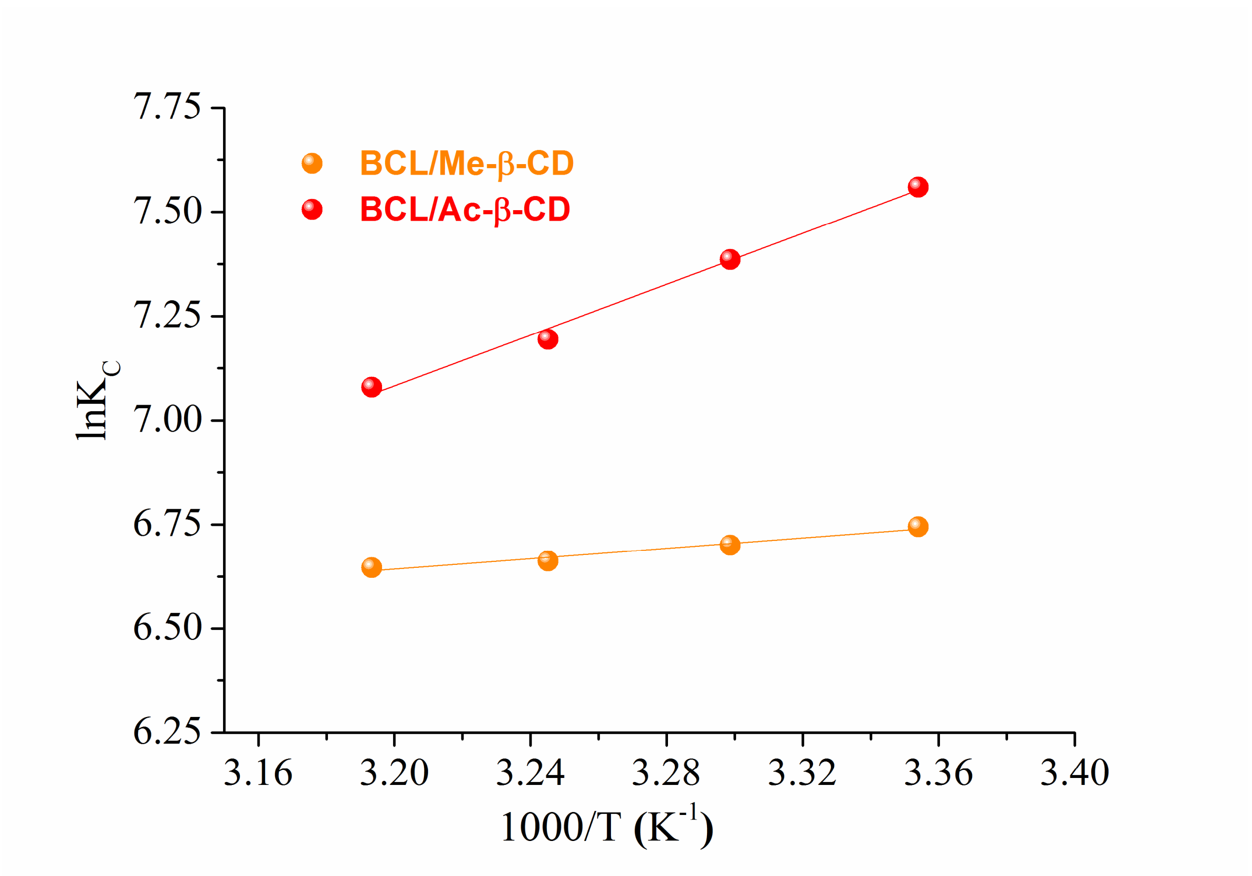

Figure S3. The temperature dependences of the stability constants for the complexation of BCL with Me-β-CD and Ac-β-CD.

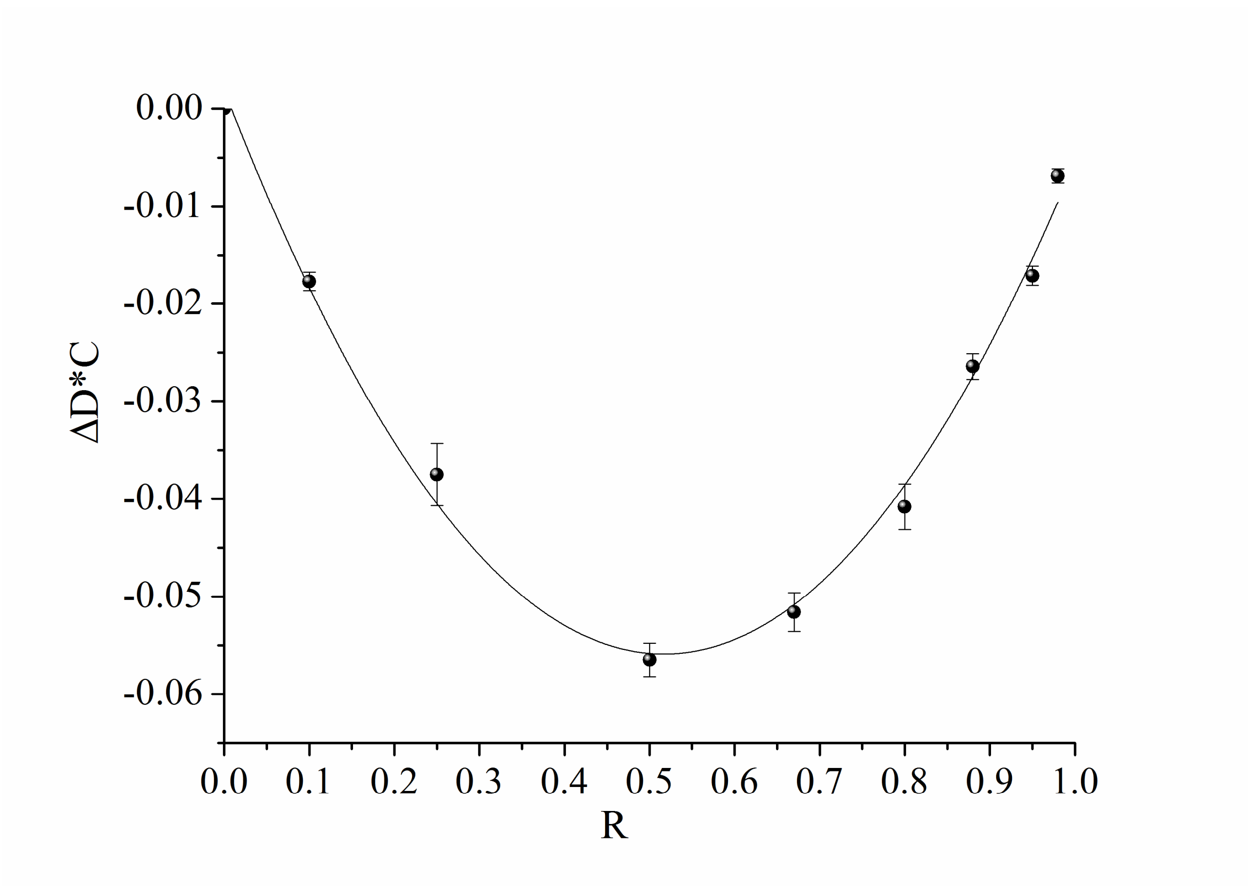

Figure S4. Job's plot for BCL/Ac-β-CD inclusion complex at 298.15 K and pH 6.8.

Table S1. BCL experimental solubility (S_2) in buffer pH 6.8 with different Me-β-CD and Ac-β-CD concentrations (S_{CD}) in the temperature range of 298.15 - 313.15 K.

S_{CD} (M)	$S_2 \cdot 10^5$ (M)			
	298.15 K	303.15 K	308.15 K	313.15 K
		Me-β-CD		
0	0.61±0.02	0.77±0.01	0.98±0.03	1.23±0.05
0.0025	2.01±0.08	2.31±0.13	2.80±0.09	3.59±0.11
0.005	3.07±0.13	3.85±0.08	4.77±0.20	5.98±0.19
0.01	5.89±0.16	6.95±0.21	8.48±0.17	10.60±0.21
0.015	8.40±0.21	10.10±0.42	12.4±0.27	15.32±0.29
		Ac-β-CD		
0.0025	3.51±0.13	3.85±0.13	4.20±0.11	4.90±0.11
0.005	6.43±0.13	6.90±0.05	7.40±0.22	8.78±0.19
0.0075	9.36±0.16	9.95±0.11	10.70±0.17	12.14±0.21
0.01	12.20±0.20	13.06±0.23	13.85±0.17	15.60±0.19

Each solubility value represents the mean ± SD (n ≥ 3)
The standard uncertainties are u(T) = 0.15 K

Table S2. Donor solution BCL concentrations (*C*), steady state flux (*J*), and permeability coefficients (P_{app}) of BCL raw sample (BCL_raw), BCL physical mixture (BCL/Ac-β-CD_pm) and ground complex (BCL/Ac-β-CD_gr) with Ac-β-CD, 37 °C.

Drug	C (M)	*J* (μmol·cm^{-2}·sec^{-1})	P_{app} (cm·s^{-1})
BCL_raw	$1.05 \cdot 10^{-5}$	$8.47 \cdot 10^{-7}$	$(8.07 \pm 0.18) \cdot 10^{-5}$
BCL/Ac-β-CD_pm	$1.21 \cdot 10^{-5}$	$9.06 \cdot 10^{-7}$	$(7.49 \pm 0.24) \cdot 10^{-5}$
BCL/Ac-β-CD_gr	$1.45 \cdot 10^{-5}$	$8.68 \cdot 10^{-7}$	$(5.99 \pm 0.20) \cdot 10^{-5}$
